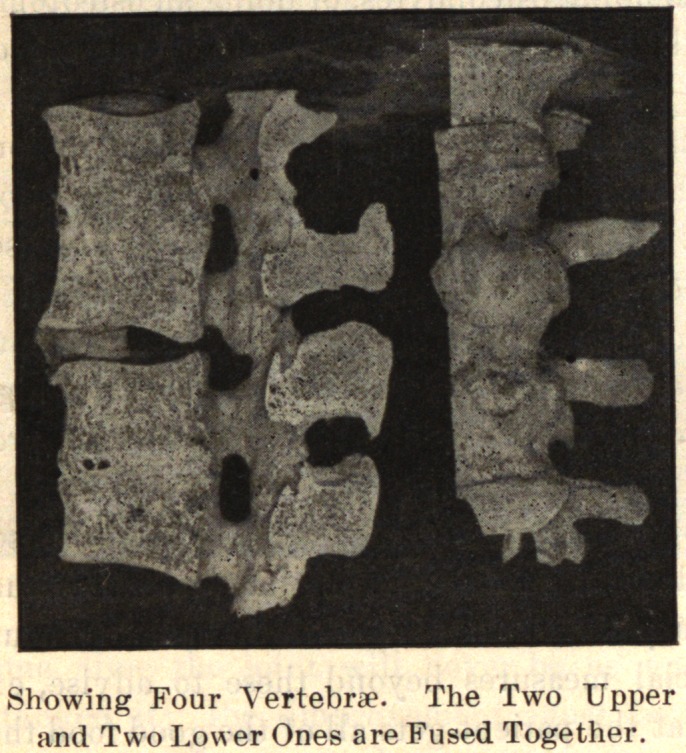# The Treatment of Osteo-Arthritis and Rheumatoid Arthritis of the Feet, Knees and Spine

**Published:** 1900-12

**Authors:** Michael Hoke

**Affiliations:** Atlanta, Ga.


### ATLANTA
### Journal-Record of Medicine.

**Successor to Atlanta Medical and Surgical Journal, Established 1855, and Southern Medical Record, Established 1870.**

VOL. II. DECEMBER, 1900. No. 9.

BERNARD WOLFF, M.D., EDITOR. M. B. HUTCHINS, M.D., BUSINESS MANAGER.

Published Monthly. Office No. 64 Marietta Street, Opposite Post-office.

### *ORIGINAL COMMUNICATIONS.*

### THE TREATMENT OF OSTEO-ARTHRITIS AND RHEUMATOID ARTHRITIS OF THE FEET, KNEES AND SPINE.

BY MICHAEL HOKE, M.D.,

ATLANTA, GA.

Observation of a large number of painful, stiff and deformed joints leads one to the opinion that, generally, not as much care is taken in the diagnosis and treatment of certain forms of joint disease as is applied to other diseases. Many of the cases, presenting no deformity, but enough pain to make walking, going up- and down-stairs, uncomfortable, came with a previously made diagnosis of rheumatism or gout, particularly where the feet or knees alone were involved; others, in which there was some little deformity, a few degrees of the twisting of the joint or of limitation of its mobility, had held aloof from care of the joints for some time, since they thought they belonged to a class of chronic rheumatics whose discomfort should be borne with cheerful resignation, since for them little or nothing could be done. Too frequently, only after obvious deformity had taken place, with its associated invalidism, was it thought that perhaps something might be done to correct the position of the crippled member.

Rounded Back from Slow Anchylosis.

One becomes convinced that what may seem to be only a little rheumatism or gout, to be treated lightly, is often to the possessor of the joint a thing of deep personal importance. The conviction that even the severest cases of the types to be spoken of, if taken in hand carefully, with a thoughtful attention to the joint's needs, may be spared much pain, and probably the trials of lameness or deformity, leads me to present to you for consideration some of the phases of rheumatoid arthritis and osteoarthritis, so often but erroneously included under the term "chronic rheumatism," to describe some of the problems they present to the orthopedic surgeon from a practical, clinical standpoint, feeling that your discussion will be of benefit not only to ourselves, but to those who in the future may present themselves for treatment and legitimate encouragement. In the vast majority of instances, if the cases are seen early, good joints will be preserved. It is true that a joint once severely injured by acute or chronic disease cannot be absolutely restored to its normal condition, but assuming that the worst crippling has occurred, if the patient be only permitted to get around with comfort, no small mercy will have been done him or her so afflicted.

Though special monographs have outlined the general medical treatment and the relation of these joints to internal medicine, the text-books on internal medicine pay only a passing attention to them. The conditions have, too, received little or no attention from the general surgical clinics on account of the time and the devotion to detailed attention necessary to obtain results. Little is to be found about them in the works on general surgery; at this time the remark is equally applicable to the text-books on orthopedics. Let me emphasize most strongly the general medical care necessary, for upon general nutrition is based a joint's recuperative power. The medical care is absolutely essential, but that alone cannot repair a mechanically faulty anatomical arrangement, relieve strain, or put motion into a stiffened joint.

The cases varied widely, both in regard to the condition in which the patients were first seen and in the extent of the involvement. In some only the hands were involved, in others the knees alone, or the spine or feet, but usually several joints were affected, the patients having waited until this was the case before coming to the clinic. The cases could be all separated into two broad classes, each different from the other and from other joint conditions. The terms "rheumatoid arthritis," used to designate one, and "osteo-arthritis," to designate the other type, are used simply because they have been employed some time. Neither has a pathological basis for its existence, for, in spite of the claims of several investigators to have found the basis of the etiology of rheumatoid, it is yet to be determined; and, too, the claim of some that osteo-arthritis is but an advanced stage of rheumatoid must be stamped as a mistake, for it occurs with its own definite change in the bone, entirely independent and distinct from rheumatoid disease. As yet, however, but for the predisposing influences we have no knowledge of the cause of osteo-arthritis or rheumatoid disease, hence only some of the gross pathological changes to be found in the two types will be mentioned, these changes by experience having been found to be sufficiently definite to identify the types and to point out the necessary treatment.

Showing Disease of Sacrum.

Osteo-arthritis is a common disease. Very many cases occur in the early twenties. Men are the most frequent sufferers. Some of the worst cases occur in young adults. Some underlying physical condition seems to be responsible for the onset of the disease, particularly loss of nerve stability, worry and the trials of too much responsibility. Often with those on life's decline, after a slight blow on the joint, the process will light up, the subsequent impairment of the joint being all out of proportion to the force of the injury sustained. One joint, or any number, may be involved. Frequently the spine is the first to suffer, the backache and sensory disturbances, with no visible evidence of vertebral diseases, leading one to that convenient side-track of diagnostic responsibility, neurasthenia.

One finds growths from the edge of the epiphyseal cartilage, which by ossification become bony nodules. The tendon and ligamentous attachments around the joint may present osseous spurs growing out from the bone as a base. The osteophytes occur on the dorsal or lateral aspect of the joint, so that the deformity, if there be any, is always one of flexion or lateral deviation, or a combination of flexion and lateral deviation, due to the mechanical presence of the nodules. The new-formed bone is always produced at the site of ligamentous attachments. In a case which recently came under my observation, in which the spine, fingers of both hands and left heel were involved, referred to me by Dr. Noble, there was a history of a bony nodule having been removed from the vagina two years ago. Dr. Noble tells me that there was spasm of the pelvic muscles and shortening of the great sciatic ligament. It is inferred that the bony nodule removed was taken from the bony attachment of this ligament. So far as I know, this is the only case in which the disease has given pelvic trouble. The type of node described by Heberden in 1804 is characteristic.

The disease is not necessarily chronic. Coincident with the involvement of the joint cartilage, there may be fluid in the joint, simulating an acute rheumatic swelling; particularly is this true with the knee-joint.

In the feet, the most common sites of the disease are the metatarso-phalangeal articulation of the great toe and the points of attachment of the plantar fascia and tendo achillis to the os calcis. Bony spurs may also be found from the edge of the articulating cartilages of the tarsal bones.

In the spine, the process usually begins anteriorly and extends up and down along the anterior and lateral ligaments. It may involve one or several vertebræ. One side alone may be involved, or one segment on one side and another segment on the opposite side. The process takes place along the ligaments. The bodies of the vertebræ may be fused together by the coalescence of the deposit on adjacent vertebræ.

If the process be confined to the side, limitation of lateral movement is seen in the early stage of the disease. Later, with complete development of the disease, there is necessarily greatly limited motion or a fixed position of the spine. If the spine curves in the development of the disease it is permanent. The curve may not be fixed in one direction, but different segments may be deviated, one to the right and the other to the left, giving a compound curve. Coincident with the bony growth from the sides of the vertebræ, the intervertebral discs absorbed are replaced by bone, so that the bodies of the spinal segment may fuse, giving a solid column.

The deformity depends partially upon the rapidity with which the anchylosis takes place. If it occur early, there is usually a straight back, if slowly, a rounded arched back.

Most serious and most important to be recognized is the extension of the disease to the transverse processes, for it is the deposition of bone around the foramina for the exit of the spinal nerves, that pinching them causes the pain and disturbances of sensation in the back and area of distribution of the nerve root pressed upon.

The clinical characteristics vary somewhat, according to the joint involved, depending in part, too, upon the use of the joint. It will be necessary to speak only of the disease as it is seen in the foot, knee and spine, to bring out the essential points of diagnosis and treatment. (The detail of a few cases quoted, illustrating the clinical picture.)

With disease of the spine, the symptoms may be all out of proportion to the extent of involvement. Often in patients over sixty years of age, with a spine rigid from atlas to sacrum, the pain is so little that it is merely regarded as one of the numerous aches of declining years; again, the pain may be severe with much muscle spasm. The seat of the pain depends upon the nerve root pressed upon; early in the disease, the pain in the back is usually referred to a definite portion of the spine, the lateral motion, flexion and extension, at this portion of the bony column, being decidedly limited by protective muscle spasm. When the pain is thoracic from pressure on the dorsal nerve roots, the condition, as regards its discomforting effect upon the patient, is exactly like intercostal neuralgia. Indeed, a large number of intercostal neuralgias are dependent upon this disease of the dorsal vertebræ. Again, there may be areas of numbness, of complete loss of sensation. In a case which I have recently seen there was at one time complete loss of sensation on the posterior half of the left side of the skull, and, which I had not seen before, an atrophy of the hypothenar group of muscles. Subsequently to this observation, sensation has returned and the muscles have regained their normal size. Often the referred pains and sensations are not met with until late in the process, the time of occurrence being dependent upon the extension of the disease to the transverse processes. Though I have not seen it, paralysis, partial or complete, may occur. If paralysis does occur it is usually unilateral. It is possible for paraplegia to develop from pressure upon the spinal chord.

Straight Back (Quick Anchylosis).

After the acute process has become limited, and the osseous tissue less vascular, the tissue shrinks, the pain markedly decreases or disappears entirely from the relief of the pressure upon the nerve roots. Improvements always take place, dependent upon the stage at which the disease is attacked and the degree of bony absorption.

The condition in the foot as regards the character of the process is the same as in the spine. The involvement of the metatarso-phalangeal articulation produces an osseous lump on the head of the metatarsal bone with a typical hallux valgus deformity, a joint painful from the recurrent attacks of acute arthritis. It is hard often to recognize the disease in the mid-tarsal bones. Patients with this type of disease usually present themselves for flat-foot; but along with the tarsal disease, the attachment of the plantar fascia and tendo achillis to the os calcis is likewise affected, giving, when the local disease is extensive, perhaps the most exquisitely sensitive heel it is possible to imagine. A spur of bone will be found attached to the calcis, passing into the fascia and tendon. Here the process requires a great deal of care and attention to overcome, owing to the pounding of the sore points in walking. The bursa behind the heel and the synovial sheath of the tendo achillis are usually inflamed, producing a very painful, fluctuating swelling behind the ankle.

In the knee-joints, in addition to the osteophytes, which form bony rims at times big enough to produce a decided knock-knee, with resultant joint strain and a valgus position of the foot, another interesting problem presents itself frequently. The bony nodes are covered by diverticula from the joint capsule; in walking, the capsule is frequently pinched and bruised; this is repeated often, so that in the end the normal villi and folds of the synovial membrane enlarge from this constant intra-arthritic traumatism; fringes or polypi are produced, equivalent, in their effect, to foreign bodies. The presence of these bodies keep up a synovitis with effusion so long as they are allowed to remain. These polypi may be palpated if by gently flexing and extending the knee one feels for them on either side of the ligamentum patellæ. The fringes slip in and out between the margins of the heads of the tibia and femur, producing a coarse crepitus.

The cases reported below, selected from a number observed and treated, will, I think, give a good clinical idea of the conditions above mentioned.

CASE 1. A. H., Male. Twenty-four years old. Clerk. His work required him to stand all day. Large frame; anemic; hemoglobin

70 per cent.; no appetite. The patient walked around the room on his toes, leaning heavily upon the chairs and other furniture. He could not stand without support. When walking with the aid of canes, one in each hand, the gait was shuffling, the knees flexed and shaking, the feet dragged along the floor, the canes bearing the body's weight almost entirely; the heels were exquisitely tender, the slightest tap causing great pain; the pain great in the instep, ball of the toes, at the attachment of the tibialis anticus and along the course of the tendons of the peronei. The tissues were puffed up behind the ankle; the arches of the feet were almost flat; there was excessive pronation; the ankle rolled in; there was some spasm of the peronei, limited dorsal flexion at the ankle, due to shortening of the tendo achillis, and some abduction of the front part of the foot, due to the spasm of the peronei. Any attempt to overcome the abduction was very painful; the feet were stiff in this position, the legs much atrophied from disuse; the muscles were flabby; there was pain in the calf of both legs, in both knees, hips, and in the lumbar spine, as is so frequently seen as a result of a pronated position of the foot, or inward rolling of the ankle. There was, too, some pain in the cervical region of the spine and occiput, which in the preceding two months has been constant; the patient had completely lost his nerve, believing that he was a cripple for life.

The X-ray showed a spur of bone at the attachment of the plantar fascia to the os calcis. It was not large, but this, with its associated periostitis and the inflammation in the soft tissues, explained the great pain in the heel. Particularly interesting in this X-ray was the dropping of the scaphoid, the key-bone to the inner longitudinal arch, below the level of the internal cuneiform bone and the astragalus. This flat foot and pronated position with stretched tarsal ligaments explained the pain in the ball of the foot, instep, legs and back. No X-ray was taken of the neck, but undoubtedly, the process was beginning in the ligaments in this portion of the spine, as the symptoms and the limitation of motion associated with the disease in the foot, pointed to this condition.

The feet were put up in plaster of paris in the corrected position for two weeks, cast was then taken and a foot-plate fitted so that it overcame the spasm of the peronei, the abduction of the front part of the foot, preserved the correct position of the arches, and held the foot so that the heel bore little of the body weight in standing and walking. Exercises were given to strengthen all the muscles of the legs, tonics were administered, and the diet forced. The case was severe, much additional detailed attention, daily for a while, and then every two or three days, strapping, bandaging and re-adjusting the plate to fit the changing tarsal arch was necessary. In three months' time the patient was able to walk two miles with very little difficulty; he had gained in weight, strength and spirit. The leg and lumbar pain disappeared with the correction of the foot, and the disappearance of the pain in the neck and occiput came gradually with the limitation of the process in the neck; a stiff neck was prevented. His ultimate perfect recovery is only a question of time, wearing suitable plates and preserving good general health. Exacerbations will undoubtedly occur with heel and tar-

Involvement of Whole Spine—Laterally.

sal pain if from worry, too much work or other causes, his general health should give way, but the process, when this takes place, can be limited to a short duration. This case illustrates the greatest severity of the disease that I have seen in the foot. Usually patients with the process in the foot are somewhat crippled, have a painful heel and tarsus, but are able to get around with discomfort, it is true, but with only a limp.

CASE 2. Mrs. B. Five years ago her husband died, leaving her to provide for herself and four children. She is a seamstress. Two years ago the disease began in the fingers with pain, swelling and stiffening of the distal phalangeal joint of the second and fourth fingers of the left hand. She kept at her work in the same surroundings and circumstances. Shortly afterwards the fingers of the right hand became involved, her general health became worse. The spine was then attacked. The pain was in the back of the neck, head, between the shoulders and in the left side. This pain was not very severe, but constant and troublesome; then the feet were involved, particularly the right foot, this incapacitated her for long standing, walking, and interfered with her work on the machine.

The fingers presented Heberden's nodes, the spine in the cervival regions was anchylosed, except between the atlas and the skull. The dorsal region was likewise rigid, but there was some motion possible in the lumbar region. The back had a slight forward arch but was comparatively straight. The disease had developed extensively, but luckily the nerve roots had only slightly felt its effect. The disease was quiescent when she came to the clinic. The foot was very painful on account of its valgus position and muscle spasm. She was put on tonics, given a two months' rest, fed high, the foot manipulated three times a week, plates applied to overcome the valgus position. In four weeks the foot was comfortable and the patient became strong. At the end of her two months' rest she was able to resume her work with comfort. She will always have a stiff spine. An attempt to give greater mobility would fail and light up the acute process again. No jacket was applied. When the anchylosis is complete and the disease quiescent there is no need for immobilization, the problem being one of nutrition and development of vitality.

CASE 3. W. B., male; teamster; aged sixty-four. He had bad pains in the back, "chronic lumbago" for five months; he worked on, but with much discomfort and pain. The back was arched forward a little; he was unable to straighten up. Lateral motion of the spine produced much pain; it was limited chiefly toward the right. There was muscle spasm on attempting to flex, extend or move the spine laterally. No bony prominence could be detected. The vertebral joints alone were affected. A plaster jacket was applied, the usual general medical treatment was prescribed. The jacket was removed in two weeks; more motion was possible in the column; the degree of pain much lessened; the jacket was reapplied and at the end of the following two weeks there was greater improvement, meaning by that, greater mobility in the spinal column and less pain. A leather jacket was fitted and worn with perfect comfort. Eventually, he will be able to discard the jacket, good motion will be preserved in the spine. This case illustrates the limitation of the acute stage of the disease, which may be often accomplished if the disease be taken hold of in time. The increased motion in the spine was due to the quieting down of the acute process and the disappearance of muscle spasm.

The class of cases included under the term "rheumatoid arthritis" present a different picture from the osteo-arthritic cases. The latter is characterized chiefly by bony hyperplasia; the former, on the contrary, shows an atrophy of the epiphyseal cartilage and an extensive change in the periarticular tissues seen in no other joint disease. It, too, may be acute or chronic. Young adults are the worst victims. The disease is common in the old. In the acute stage the joint is enlarged, assuming a characteristic spindle shape, the swelling being chiefly in the periarticular structures. Usually there is very little fusion in the joint capsule. The X-ray shows no bony enlargement. The soft structures around the joint feel boggy as if macerated. In addition to the hyperplastic thickening, the tendons may become so disorganized that they lose their identity and like the joint ligaments become simply a part of a mass of dense periarticular fibrous tissue. The muscles, both distal to and proximal to the joint, atrophy. The extensors seem most frequently to be involved; the atrophy is usually rapid; the glands on the proximal side show an hyperplasia. There is not much fever in true rheumatoid disease; the temperature does not go over 102.5. The epiphyseal cartilage softens, becomes eroded in areas; this osteoporosis is followed by osteo-sclerosis, producing an eburnated smooth area on the end of the bone. The atrophy of epiphyseal cartilage is characteristic of the latter. When the acute stage is passed, the joint is smaller than normal. There is one exception to this rule when the joint effusion has not been absorbed. Two things are most often responsible for the continued presence of the fluid. If, as sometimes happens, there be a little blood in the effusion, coagula are formed, sometimes one inch long by three quarters of an inch wide. These coagula may break down or become a part of a number of dendritic processes attached to the synovial capsule. Again, if from the thickening of the joint capsule the villi are enlarged, as happens at times in the osteo-arthritic process, they are in effect equivalent to foreign bodies. There is this difference in the causation of the polypi in osteo-arthritis and rheumatoid arthritis; in the former, the enlargement of the villi is not due to any disease of the capsule, but simply to the swelling and thickening incident to constant bruising from being pinched by the osteophytes on the heads of the articulating bone; in the latter disease, there is no mechanical injury by osteophytes, but primary infiltration of the villi; the capsule takes part in the change in the tissues due to rheumatoid disease. As long as these polypi and coagula remain in the joint irritation is kept up, the joint is painful and the fluid remains. The condition produces, by use of the joint, a constant intra-arthritic traumatism to an already impaired joint.

Often the hard brawny feel of the joint to the palpating finger, the limitation of motion, the muscle spasm, the apparently enlarged tuberosity of the tibia or apparently thickened condyle of the femur, closely simulate a tubercular process. There seems to be a difference, however, in the muscle spasm in tubercular disease and rheumatoid disease. In the former there is a tightening of the muscles which not even the gentlest manipulation can coax to give away and permit motion, in flexion or extension, beyond what at first seems to be the limit in the individual case. In the latter condition, though the joint may at first permit no flexion, by careful, gentle manipulation, the spastic muscles may be induced to let up in their tension; the brawny thickening, as hard as bone itself, will be shown by the X-ray not to be bone disease, but thickening of the periarticular soft structures. Hernias from the capsule into the surrounding tissues occur. These, by occlusion of the communicating process with retention of their fluid contents form tense swellings on the side of the joint, which, from their inelasticity, due to the thickening, may give the impression of condyloid or tuberrous bony prominences. These constitute some of the conditions to be carefully looked for and to be borne in mind in the treatment of the diseased joint.

In the large joints, particularly the knees, which so often call for care, the deformity is always one of flexion, to which is usually added a partial subluxation backwards of the head of the tibia and torsion of both bones of the leg. Walking with a joint affected like this is nearly always productive of pain in the joints above and below, even if there be no rheumatoid disease of the latter. The joints are all most accurately constructed upon mechanical principles. A change in the relation of the heads of the bones of the knee changes the line of action of the body weight, throws unnatural strain upon hip, knee and tarsal joints, upon the ligments and upon the muscles whose tonicity and balance control their proper action. As a result of this, even if the disease be in the knee-joint, the feet may present interesting problems to be most carefully looked after. Often knee pain and hip pain and foot pain, with chronic disease only in the knee, is absolutely relieved by using a foot plate that shifts the line of action of the body weight. The philosophy of this is simply that before the use of the plate the body weight acts in a way to strain the knee, which is overcome by keeping the position shifted to one of comfort by the constant wearing of a foot-plate.

CASE 1. J. McM., nineteen years old. There was no history of rheumatism or tuberculosis in the family. The disease was in the right knee. Two months before entrance to the clinic the disease came on. There was no injury. The right knee swelled up one night and was painful. He did not stay in bed with it the next day. For several days he reclined on a lounge most of the day, and the rest of the time limped with a cane; he had very little fever, no sweats. There was very little effusion into the joint; he went to another clinic. A diagnosis of tuberculosis of the knee was made and the joint incased in plaster of paris in a straight position. When first seen the knee could be flexed passively through five degrees; no effusion into the joint; there was extensive atrophy above the joint; the pain was severe on manipulation. There did not seem to be the absolute muscle spasm seen in joint tuberculosis. This, with the character of the onset, the slight temperature, the little effusion, the enlarged spindle-shaped joint, suggested rheumatoid. The X-ray showed the thickening not to be in the bone but in the soft tissues. There was no bone disease at all, the rheumatoid disease having affected only the soft structures. The patient was etherized, the joint flexed and then extended with gradually increasing force, so as not to tear loose cartilage with the breaking up of the adhesions, as may be done by too sudden and forceful manipulations. The leg was put in plaster. Subsequently measures to be spoken of later were carried out. The joint recovered with 45 degrees flexion; this will increase in time with use. This case illustrates the great care that should be taken in a doubtful case. It had been called tuberculosis in another clinic; the treatment of the case for rheumatoid was so radically different from that for a tubercular joint that it meant much to this patient in its effect upon his life for a few years.

Showing New-formed Bone, as if Molten Bone had been Poured over these Vertebræ.

CASE 2. Miss H., twenty-six years old. She had chronic rheumatoid disease of both hands and knees; several of the fingers were hyperextended, showing the atrophy of the joint cartilage. Both knees were involved; there was fluid in both joints. The knee-joints had been swollen somewhat for ten months. They could be flexed through 60 degrees and completely extended, but these motions always brought pain. She had a good deal of pain when walking, particularly when going up- or down-stairs or climbing a hill. At times the joints would swell considerably, when she would have to go to bed; then the swelling would partially subside and she would be able to get about, but with discomfort. Polypi were palpated. She was put on tonics and forced diet, and kept in bed for awhile with the knees bandaged; knees were then opened; the polypi studded the synovial membrane. What is particularly interesting in this case is the fact that all of the polypi big enough to give trouble were removed from the left knee; the right knee was not so thoroughly cleaned. Subsequent to the operation the left knee became strong and gave no trouble, but the right continued to swell at times. The synovial dendrites left in the right knee continued to keep up some pain and effusion, though the joint was improved by the partial removal of them. She gained weight, strength and general health by the operation. There seemed to be a general effect on the other joints, too, though none were operated upon but the knees. The improvement of the other joints was due to the improvement of her general health, which had been impaired chiefly by the condition of the knees. The improvement of the other joints consisted in the ability to use them without pain. Of course the hyperextension of the fingers could not be remedied. This case illustrates beautifully what may be accomplished for the joints by operation when the involvement seems to be chiefly synovial, and, too, it demonstrates the effect of the polypi, the result to be obtained by their removal and the result of leaving them in the joint.

CASE 3. W. R. T., sixty years old. Subject of rheumatoid disease twenty years. It had progressed so that all of the joints but the hips were involved; the knees were fixed in 30 degrees flexion, both legs were rotated outward; the feet were in the valgus position. The knee-joints were manipulated two years ago with the genuclast, hot-air baths were given the joints; the diet was forced; there was a studious avoidance of salicylates and other depressing drugs. The patient was seen in the spring; he was still a cripple, but able to get around with the assistance of caliper-splints. From being an absolutely helpless, bedridden invalid, unable by himself to attend to the personal necessities of daily life, he was able to get around fairly well and with comfort. This case illustrates that by manipulation and the assistance of apparatus usually the worst cripple may be helped somewhat.

Coming finally to the general consideration of these joints one must accentuate several things. The types of disease spoken of are distinct entities; they are not chronic rheumatism, nor are they to be called arthritis deformans. The processes in the two types are separate and distinct; they have to be treated differently. In handling a case the type of the disease must be recognized; it must be attacked in its earliest stages to obtain the best results, whether it be rheumatoid arthritis or osteo-arthritis. Neither type should ever receive antirheumatic treatment, for that means depressing drugs and curtailment of the food supply. Just the opposite should be done in these two diseases; everything should be done to build up the patient's general health, and nothing permitted which tends to lower vitality. In each individual case meet the special surgical need, devise and apply the special apparatus that seems requisite to relieve the joint of a great deal of its weight-bearing function and yet permit it to be used. When a disease has lasted some time the joint will never be as good as before the disease attacked it; the improvement must be in the degrees. The process may be arrested after it has attacked only one or a few joints, and then that one joint or the few enabled to so perform their function that the patient may be spared the usual trial of the cripple. In the majority of the cases a movable joint may be obtained, but the degree depends upon the joint involved, the altered relation, the heads of the bones, upon the condition of the ligaments and tendons, upon the form and extent of the anchylosis. The two types require entirely different modes of treatment.

Showing Bony Lipping of the Vertebral Bodies.

In the osteo-arthritic cases with cartilage and bone hypertrophy the normal use of the joint, from the fact that the cartilaginous and bony growths impinge upon one another, produces a constant irritation and traumatism, tending to stimulate the continued pro-[illegible]ction of bone with progressive impairment of the joint. It is, consequently, to be expected that any forcible manipulation will produce an additional injury to that resulting from natural exercise. Forcible manipulation may result in lighting up the process, or it may break off an osteophyte, adding the complication of a foreign body in the joint. Depending upon whether the condition be acute, sub-acute or quiescent, one has in general to consider the question of absolute immobilization or of allowing partial mobility by a splint, which permits this but takes some of the work from the joint, of removing offending osteophytes, of doing an osteotomy for correction of a lateral deviation of the bone, and of the use of foot plates.

In conjunction with the local care of the joint, of great importance is the solving of the problem of each individual's digestion and assimilation of food. A joint cannot regain its power and usefulness unless the muscles whose tendons are attached around it are brought up to normal tonicity and strength. Hot air, stimulating baths not prolonged long enough or with sufficiently high temperature to produce depression, are of great assistance in promoting local circulation and are soothing in effect.

Acute exacerbations of rheumatoid disease should receive absolute rest, forced diet and the usual non-specific but tonic medicinal treatment conducive to a patient's comfort while in bed. In acute attacks there are no special measures beyond these to advise, except to forcibly insist that the patient gets all of the good food that can be conveniently ingested, air and no salicylates. After the acute stage is past the problem is to regain motion in a stiff and probably deformed joint. The anchylosis may be partial or complete. In the vast majority of cases it is fibrous in character. There are no bony outgrowths, as in osteo-arthritis, to do injury to the joint by forcible manipulation. The patient should be thoroughly etherized. The joint is put through complete flexion and extension once. The application of force should be gradual. There is danger to the joint if the correcting force be suddenly applied. If the manipulation be thus carefully done, the chances of exciting an acute process are minimized. If the knee is the joint to be manipulated before the correcting force is applied, the patella should be worked loose. The joint is then immobilized in a plaster of paris dressing. In five or six days the plaster is cut down, the joint is gently flexed once through a few degrees. The patient is made to do this voluntarily twice a day. The amount of motion is very little at first, but it increases gradually. At this time much discomfort is alleviated and the muscle spasm decreased by the hot-air bath.

Eventually, in three or four weeks probably—sometimes earlier, sometimes later—the muscle spasm will have disappeared. The plaster dressing should not be taken off until this is the case. When this is the case, what has been passive motion should now become voluntary; the joint and muscles should be given more work, muscles above and below the joint should be massaged, and the patient allowed to walk with caliper-splints and crutches. Strength gradually returns, so that the crutch may be discarded; only with the worst cases is it necessary for the patient to use caliper-splints or crutches for years.

Showing Four Vertebræ. The Two Upper and Two Lower Ones are Fused Together.

If there be partial subluxation of the head of the tibia, as so often happens, it will be impossible to restore the position of the bones by the hands. Under such circumstances the genuclast must be used. This instrument is so constructed that it forces the patella from its adhesive attachment and reduces the dislocation of the head of the tibia while forced extension of the leg is being accomplished. If the disease be in the foot it is usually necessary to break up the adhesions between the tarsal bone and the ankle joint, to immobilize the foot in the corrected position in plaster of paris. Then a foot-plate is applied that will support the tarsal arches. If the disease be limited to the foot, in addition to the local care, one must not forget the importance of general medical treatment, for without toning up the system results are always delayed.

It often happens that the tendo achillis has so shortened that the dorsal flexion of the foot may not be possible to the normal degree. Under these circumstances walking cannot but throw the foot in the valgus position, keeping up the flat foot and tarsal strain, and permitting the spasm of the peronei. Tenotomy of the tendo achillis, or better, stretching by means of the Schaffer shoe, is necessary.

In conclusion, I desire to express my indebtedness to the kindness of Dr. Joel E. Goldthwait, of Boston, for the opportunity of making these studies while working in his adult orthopedic clinic at the Carney Hospital, and, too, for the privilege of reproducing the accompanying cuts, which are from enlarged photographs of pathological specimens in the Harvard Museum.

## Figures and Tables

**Figure f1:**
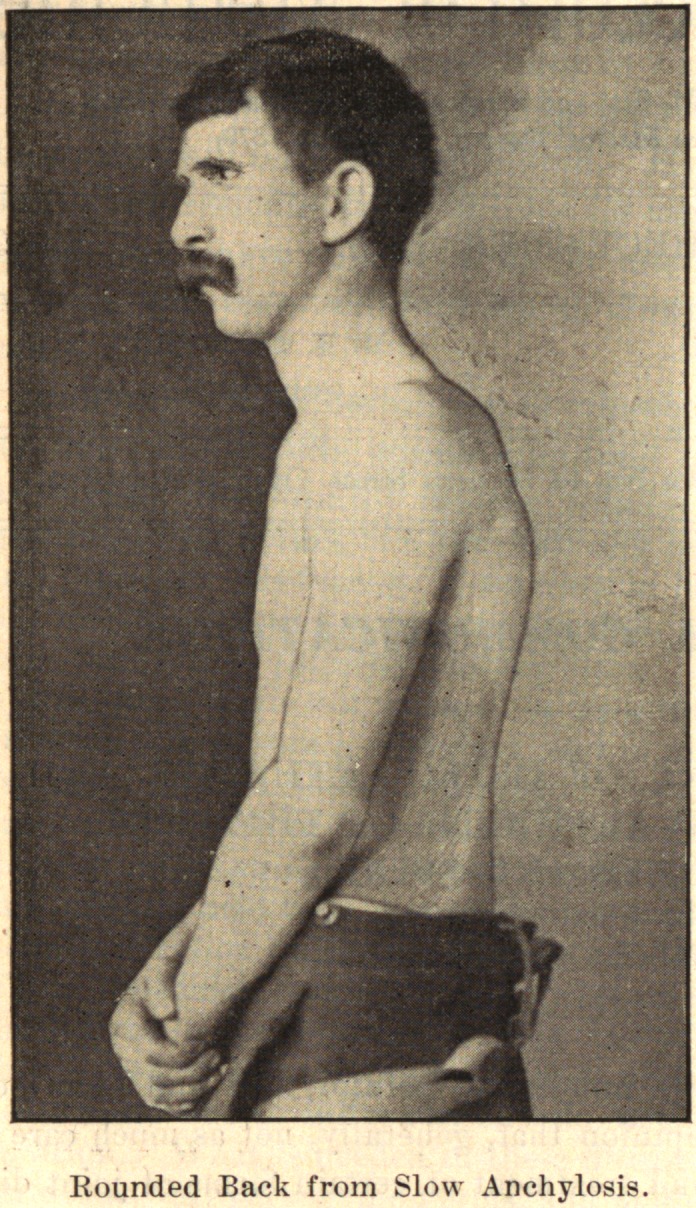


**Figure f2:**
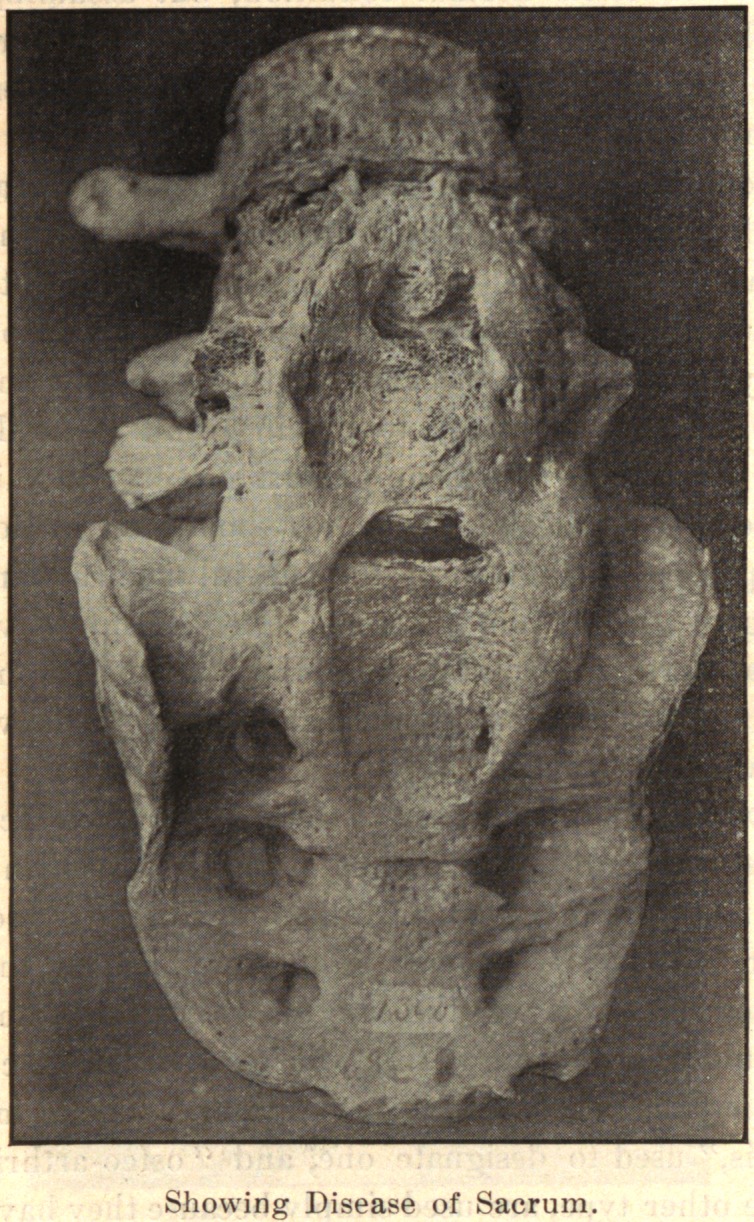


**Figure f3:**
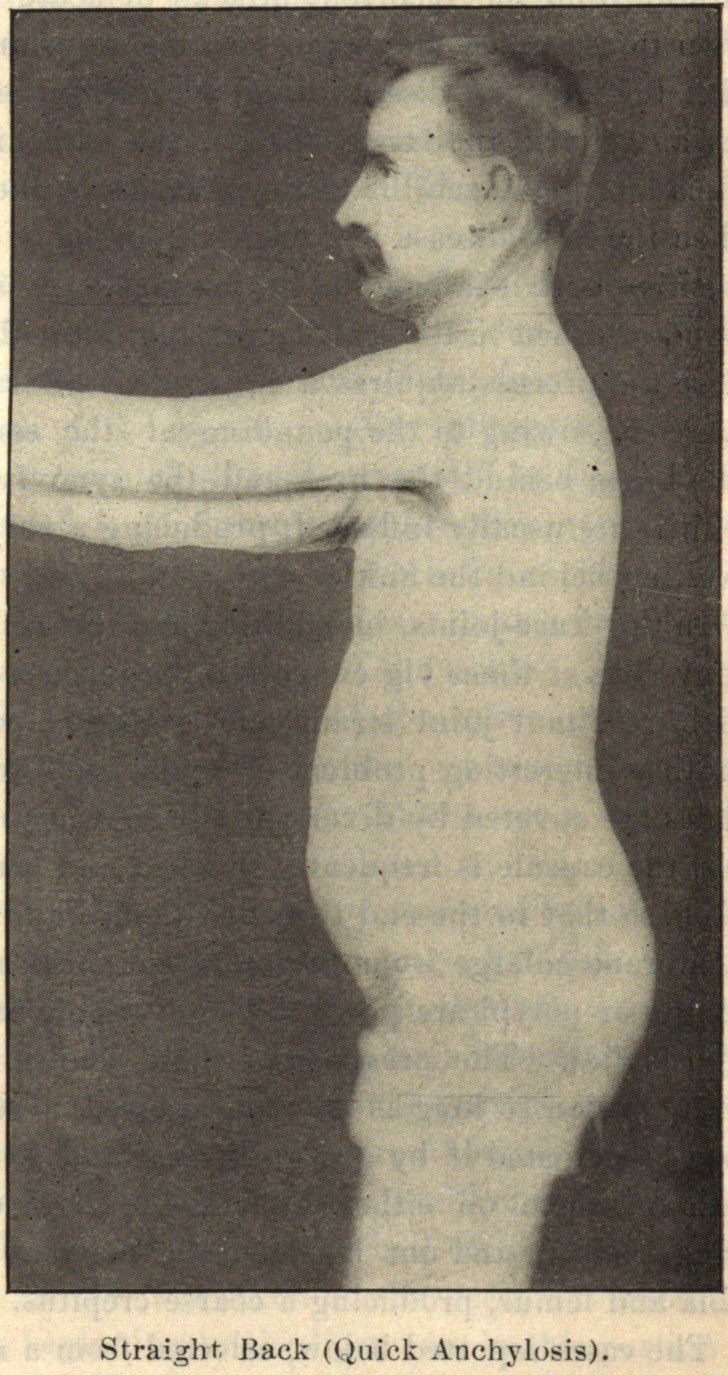


**Figure f4:**
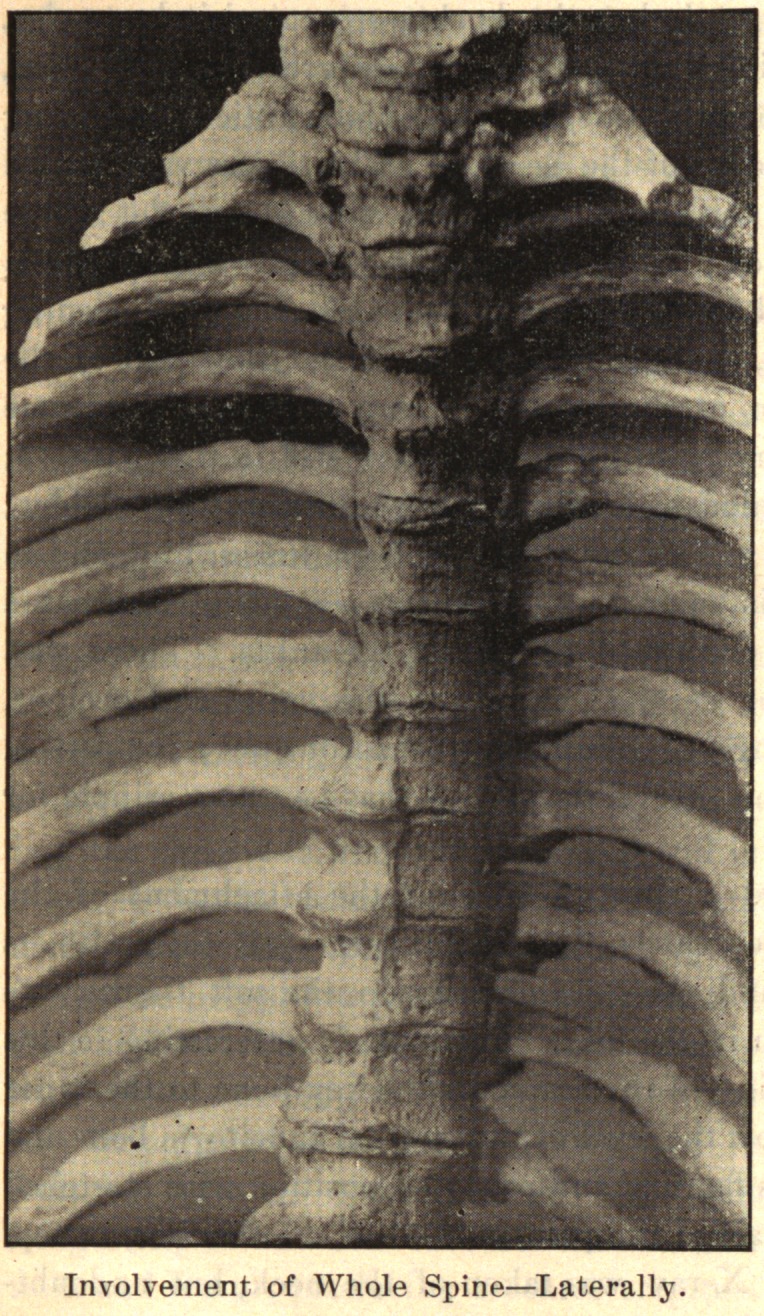


**Figure f5:**
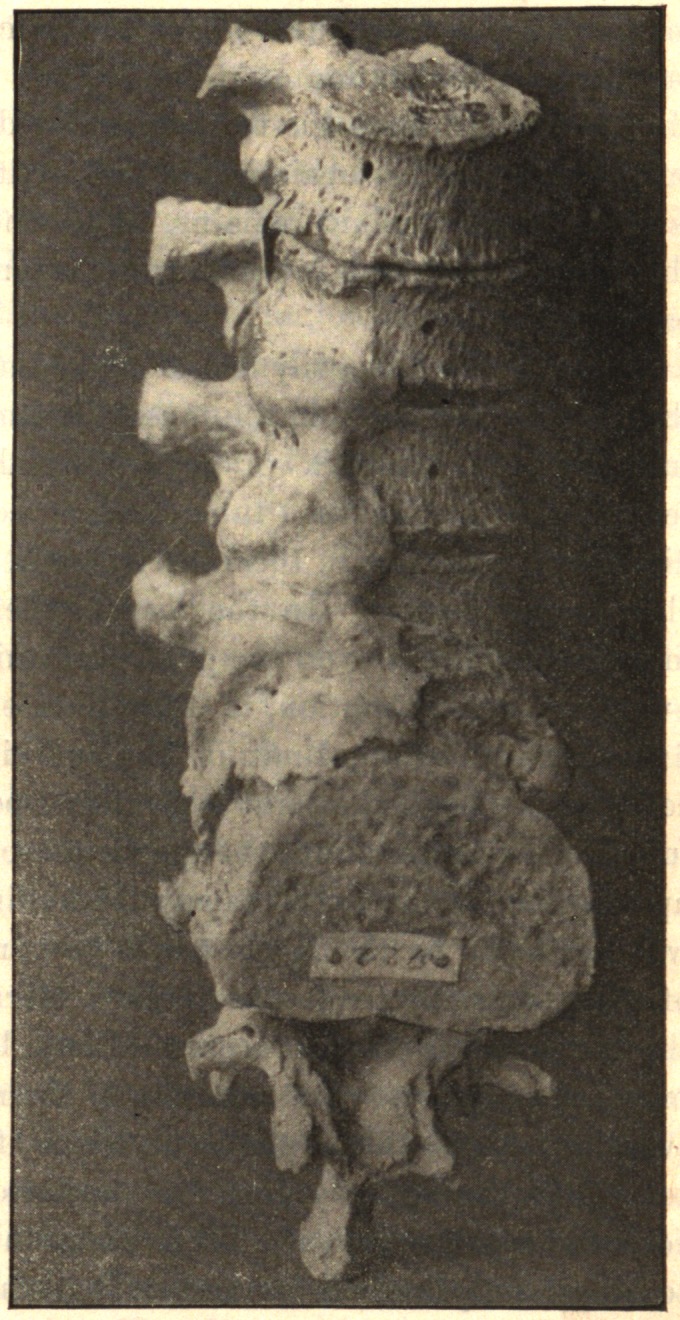


**Figure f6:**
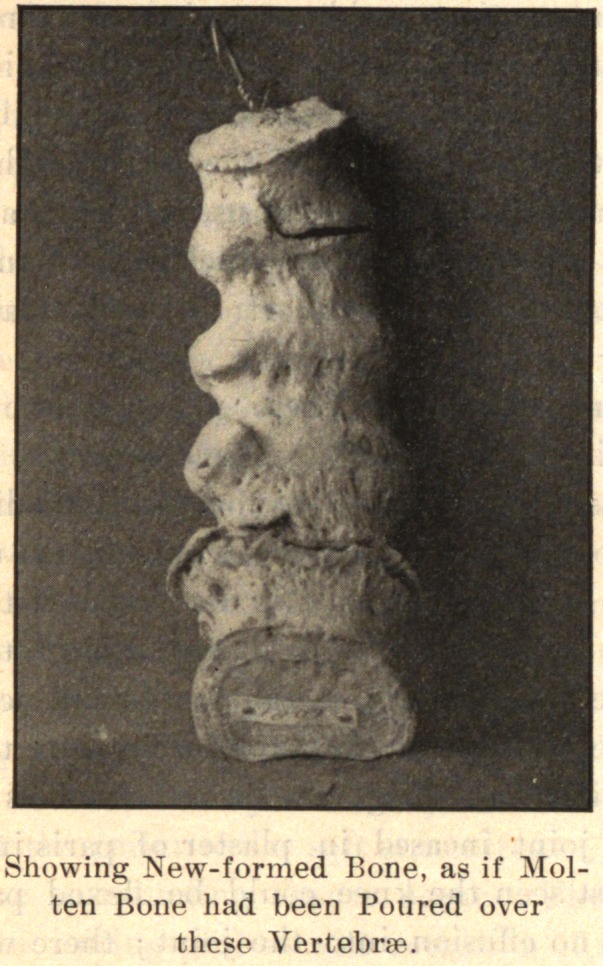


**Figure f7:**
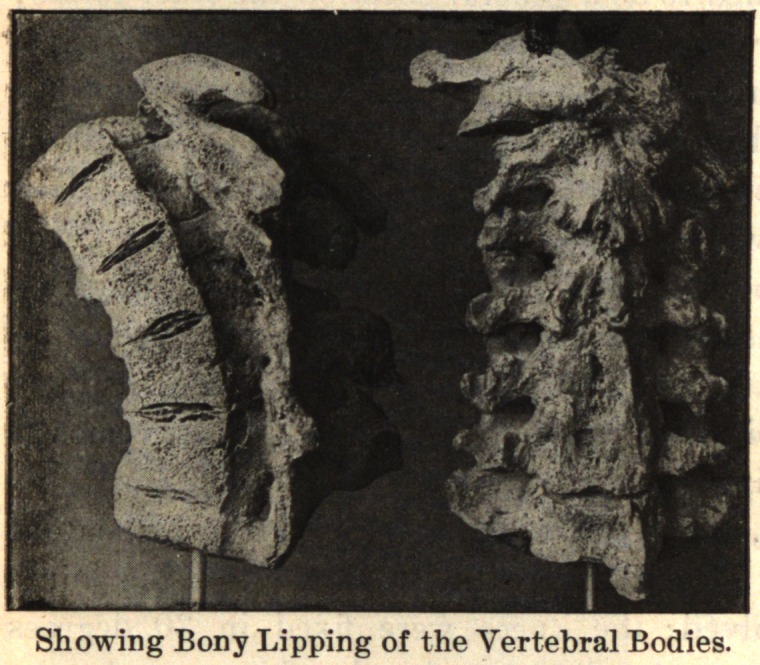


**Figure f8:**